# *Marañon* Nutshell Extract as a Carbon Steel “Green” Inhibitor in Marine Environments

**DOI:** 10.1021/acsomega.4c07363

**Published:** 2024-11-27

**Authors:** David Bonfil, Lucien Veleva, Diana Rubi Ramos-López, William Santiago González-Gómez, Gloria Ivette Bolio-López

**Affiliations:** † Applied Physics Department, Center for Research and Advances Study (CINVESTAV), Campus Merida, Merida, Yucatan 97310, Mexico; ‡ Popular University of Chontalpa, Cardenas-Huimanguillo Highway Km. 2.0, Cardenas 86500, Mexico

## Abstract

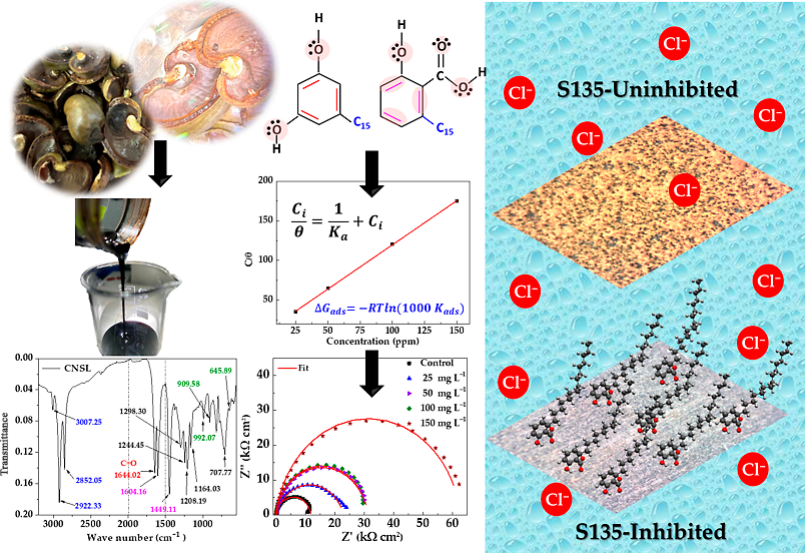

Natural *marañon* nutshell extract was obtained by mechanical compression. The extract was combined with ethanol and a nonionic surfactant, and it was labeled as EES. The EES inhibitor effect on S135 carbon steel, exposed to a simulated marine-coastal environment (SME), was deduced by mass loss measurement, adsorption isotherm, electrochemical measurements, and surface analysis. The Langmuir adsorption isotherm suggested that a monolayer of the *marañon* extract was attached by physical–chemical interaction with the steel surface. The increase in the protective efficiency of the adsorbed EES inhibitor was ascribed to the gain of the surface coverage as a function of the inhibitor concentration. It was considered an antioxidant activity of the inhibitor, attributed mostly to the Fe-ion capture by anacardic acid and the posterior ion chelation. This fact was collaborated by the negative zeta potential of the *marañon* nutshell extract, added to the SME. Electrochemical impedance spectroscopy (EIS) diagrams revealed that the steel polarization resistance (*R*_p_) increased as a function of the inhibitor concentration, while the thickness (*d*) of the Fe-oxide layer was reduced to ≈0.50 nm.

## 1 Introduction

Carbon steels (CS) are widely used materials in a variety of industries, such as oil and gas pipelines, ship structures, offshore platforms, automobiles, and construction structures, due to their good mechanical performance and low cost.^1,2^ However, when CS are exposed to acid solutions (during industrial pickling and cleaning) or marine-coastal aggressive environments, their surfaces are attacked by the corrosion process, which leads to loss of metal mass. The marine environment is characterized mainly by the presence of aggressive chloride (Cl^–^) ions, and the degradation of the CS occurs as localized corrosion, known as a pitting attack. In the maritime sector, the consequence of corrosion could result in serious economic losses and environmental complications, in the order of trillions of dollars/year, according to the NACE (National Association of Corrosion Engineers) reports.^3,4^

To mitigate the corrosion of CS, the use of corrosion inhibitors is considered as a practical, effective, and economic method.^5,6^ Many conventional organic corrosion inhibitors (containing heteroatoms of O, N, P, and S), likewise inorganic (chromates, dichromates, nitrites, nitrates, borates, arsenates, or phosphates), are nonbiodegradable and toxic compounds for the human and environmental health, which is why their use has been restricted.^7,8^ Therefore, biodegradable and nontoxic “green” corrosion inhibitors have been promoted.^9,10^ In recent studies, the benefit of extracts from plants, herbs, and shell oils, as metal corrosion inhibitors, has gained great attention in scientific research, due to the variety of bioactive compound composition.^11^ Some examples are extracts of orange peel waste,^12^ almond peel,^13^ watermelon rind,^14^ and cucumber peel,^15^ as well as cinnamon oil^16^ and aloe vera gel,^17^ among others, which have been considered as “green” corrosion inhibitors for CS exposed to acidic environments. On the other hand, extracts of tobacco,^18,20^ natural honey,^19^ aloe vera,^20^ pistachio leaves,^21^ olive leaves,^22^ cinnamon,^23^ and grapefruit^24^ have been reported with inhibitor efficiencies of ≈75–95% for steels exposed to NaCl-contaminated environments. To deduce the inhibitor mechanism, the most common methods reported are the measure of the metal mass loss (ML), adsorption isotherms, potentiodynamic polarization curves, and electrochemical impedance spectroscopy (EIS) diagrams.

The plant extracts are characterized by their phytochemical combination of flavonoids, saponins, alkaloids, tannins, glycosides, anthraquinones, and phenolic compounds, among others, which may provide an inhibitory effect, powered by the presence of aromatic rings, structure unsaturation and many molecular functional groups (–OH, –COO, –COOH, –NH_2_, –CONH_2_). These groups have electron-rich sites, prone to adsorb on metal surfaces, through electron donation and retrodonation to the vacant d-orbitals of iron (Fe) and other metal atoms.^25,26^

**Figure 1:**
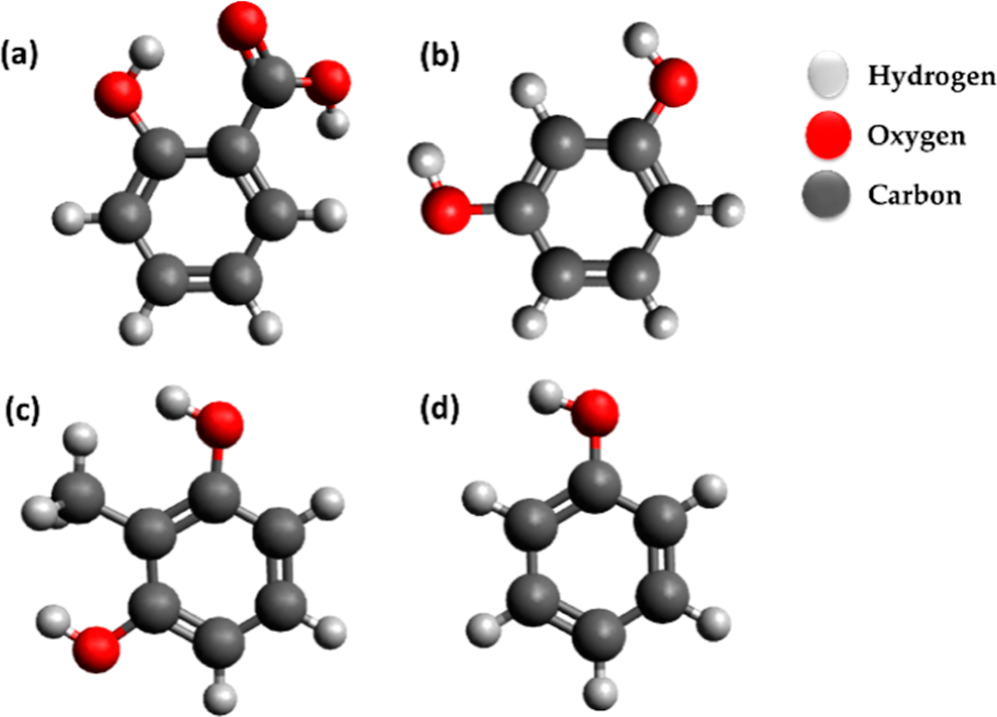
Main structures of the CNSL benzene rings in (a) anacardic acid; (b) cardol; (c) methyl cardol; (d) cardanol. Adapted by (Mgaya et al. Cashew nutshell: a potential bioresource to produce biosourced chemicals, materials and fuels. *Green Chem.***2019,***21,* 1186–1201. 10.1039/C8GC02972E).

The cashew nutshell liquid (CNSL) extract is a byproduct of cashew nuts (known as *marañon* nuts in the region of Tabasco, Mexico), classified as technical or natural CNSL.^27^ The natural CSNL is composed mainly of 60–70% anacardic acid, 10–20% cardol, 3–10% cardanol, and 2–5% methyl cardol. Meanwhile, the technical CNSL contains mostly cardanol due to the thermal decarboxylation of the anacardic acid.^28^ Such phytochemical compounds of the CNSL are known as alkylphenols or phenolic lipids, composed of an unsaturated benzene ring (Figure 1) with a 15-carbon site chain. The side chain may have partial unsaturation, as in the case of cardol, cardanol, and 2-methyl-cardol,^29^ giving these compounds corrosion inhibition characteristics.

The technical CNSL extracts have been considered as a corrosion inhibitor for carbon steel exposed to acidified fluids. The residue cardanol after the distillation of CNSL has been dissolved in polar solvents (propyl alcohol, butyl glycol, and acetylenic alcohol), and the inhibitor efficiency for the carbon steel API P110 has been evaluated in a 15% HCl solution.^30^ Thus, this residue lowered the steel corrosion rates, in which an inhibitor efficiency of ≈95% was attributed to the major content of polycondensed aromatic molecular structures, likewise considering the synergistic effect based on the addition of an acetylenic alcohol solvent. The results were supported by the ML data, potentiodynamic polarization curve records, and EIS diagrams. The inhibitor efficiency was improved by adding halides (KI).^31^ Amino-pentadecylphenols, synthesized from chemically modified technical CNSL, revealed an inhibitor efficiency of ≈97% for mild steel exposed to a 0.1 M HCl solution.^32^ On the other hand, a pretreated cashew nutshell liquid extract, with the addition of hydrazine hydrate, was considered as a corrosion inhibitor for carbon steel in 0.1 M NaOH alkaline ambient, as well as in a 3%  solution (saturated with CO_2_ gas). The inhibitor efficiency of ≈92% was obtained through potentiodynamic polarization and electrochemical impedance records, suggesting a mixed type of inhibition.^33^ The CNSL, dissolved in 15.14 mol L^–1^ ethanol, was reported as a corrosion inhibitor for aluminum, stainless steel, and mild steel, exposed to a 3.5%  solution; the inhibitor efficiency for mild steel was ≈77.5%.^34^ The studied technical CNSL extracts have been obtained by the pretreatment of the extract, such as distillation (to separate the cardanol) or by the chemical modification of the extract.

In this study, the natural extract of *marañon* (*Anacardium occidentale*) fruit nutshell, obtained by mechanical compression, was proposed as a corrosion inhibitor for the carbon steel S135, exposed to a simulated marine-coastal environment (SME), when an aqueous layer at 100% air humidity was formed on the metal surface. It was assumed that the extract might have a high content of anacardic acid, which would be beneficial to the inhibitor effect.

The composition of the *marañon* extract was characterized by Fourier transform infrared (FTIR) spectroscopy. The zeta potential (surface charge) of the molecule present in the *marañon* extract was measured. The free corrosion potential (OCP) of the steel was monitored during its immersion in the SME as a function of different inhibitor concentrations. The adsorption isotherms were obtained to propose the inhibitor mechanism. EIS diagrams were obtained to reveal the specific characteristics of the steel–solution interface. After exposure to the SME, the ML of the steel was measured, and the inhibitor efficiency was determined. The surface of the steel exposed to the SME was characterized by X-ray photoelectron spectroscopy (XPS) and an optical microscope.

## 2 Experimental Section

### 2.1 *Marañon* Nutshell Liquid Extraction and Characterization

The raw nuts of *marañon* (*A. occidentale*) were collected from the region of Huimanguillo, Tabasco state of Mexico. The nutshells were removed from the fruit and then were twice washed with distilled water (18.2 ) and dried at 38 °C for 5 days. The nutshell extraction was assisted by a 4 ton load of mechanical compression. The characterization of the extracted functional groups was assisted by FTIR spectroscopy (Nicolet iS 50, Thermo Fisher Scientific, USA), using the attenuated total reflection technique in the range from 4000 to 500 cm^–1^. Due to the low solubility of the nutshell extract in water, ethanol and the nonionic surfactant “Tween 20” (Accion Quimica AQ, Mexico) were added (ratio 1:2:2) and the liquid extract solution was labeled as “EES”. The zeta potential, as a surface charge of the EES inhibitor molecules added to the SME, was measured by a Zetasizer (NANO ZEN 3600, Malvern Instruments Ltd., UK).

### 2.2 Carbon Steel S135

According to the producer (PICO Energy, Mexico), the nominal composition (wt %) of laminated steel is 0.20 C, 0.86 Cr, 0.403 Ni, 0.54 Mn, 0.85 Mo, 0.24 Si, 0.06 Cu, 0.004 S, 0.007 P, and the balance Fe. Rectangular samples (20 × 20 × 3 mm) were wet abraded with SiC paper up to 600 grits, using ethanol as the lubricant, then sonicated (Branson 1510, Branson Ultrasonics Co., USA) in ethanol for 10 min, dried at 21 °C, and stored in sealed containers.

### 2.3 Immersion Test

The SME (pH = 7.74) was prepared with analytical-grade reagents, dissolved in ultrapure deionized water (18.2 MΩ cm^2^): 5.84 g L^–1^ NaCl, 4.09 g L^–1^ Na_2_SO_4_, and 0.20 g L^–1^ NaHCO_3_.^35^ Concentrations of 25, 50, 100, and 150 mg L^–1^ of the EES inhibitor were introduced in 20 mL of the SME solution, in independent containers, according to ASTM G31-12a,^36^ immersing the samples of S135 steel for 24 h (at 21 °C). At the end of the immersion test, the samples were withdrawn, washed, dried at room temperature, and stored. To measure the steel ML, the formed layer on the steel surface was removed by pickling, according to ASTM G1-03,^37^ using a solution of 50% HCl and hexamethylenetetramine. The ML was calculated as the difference between the initial and final steel mass, measured in triplicate by an analytical balance (VE-204, Velab, MEX).

The steel surface coverage (θ) by the EES and its inhibitor efficiency (% IE_ML_) were calculated from the average of triplicated measurements, according to eqs 1 and 2
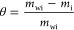
1
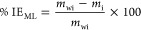
2where *m*_i_ and *m*_wi_ are the ML (mg) of the tested steel samples tested with and without the inhibitor, respectively, after the removal of the formed layers.

### 2.4 Surface Characterization

The surface of steel samples withdrawn after 24 h of exposure to the SME, in the presence and absence of the inhibitor, was analyzed by an optical microscope (Scope A1, ZEISS-AXIO, DEU). The corrosion product composition was suggested by XPS (K-Alpha, Thermo Scientific, USA), after 20 s of surface erosion with a scanning Ar-ion gun.

### 2.5 Electrochemical Measurements

Electrochemical measurements were performed with a cell configuration of three electrodes (inside a Faraday cage): the saturated calomel electrode (SCE, CH Instruments Inc., USA) as a reference, platinum mesh as an auxiliary, and the steel S135 samples as the working electrodes (0.8 cm^2^), respectively, connected to a potentiostat/galvanostat (Interface-1000E/ZRA, Gamry instruments, USA). The change of the OCP, known as free metal corrosion potential, was registered after 24 h of immersion of the steel samples in the SME solution, in the absence and presence of EES as an inhibitor. EIS measurements were performed from the 100 kHz to 10 mHz frequency range, with an alternating current signal of ±10 mV at OCP and 10 data points per decade of sampling size. V. 7.1 Gamry Echem Analyst software (Philadelphia, PA, USA) was used to analyze the EIS data. The inhibitor efficiency, based on the EIS data (% IE_EIS_), was calculated according to eq 3
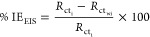
3where *R*_ct_i__ is the charge transfer resistance of the inhibited metal surface and *R*_ct_wi__ is the charge transfer resistance in the absence of an inhibitor.^38^

## 3 Results and Discussion

### 3.1 FTIR Analysis of the *Marañon* Nutshell Extract

**Figure 2:**
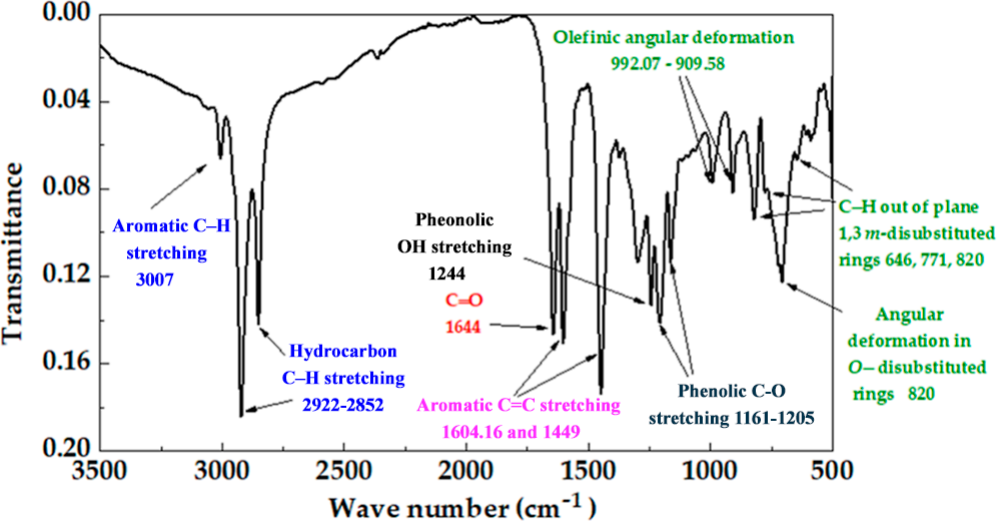
FTIR spectrum of the *marañon* nutshell extract.

Figure 2 presents the FTIR spectrum of the natural *marañon* nutshell extract; the characteristics bands were associated as follows: C–H aromatic stretching (at 3007.25 cm^–1^), corresponding to the phenol structure; C–H hydrocarbon stretching (at 2922 cm^–1^ and 2852 cm^–1^), ascribed to the alkyl side chain; C=O (at 1644 cm^–1^), associated with the carboxylic group of anacardic acid; C=C aromatic stretching, corresponding to the phenolic rings;^34,39^ and C–O phenolic stretching (at 1162 and 1205 cm^–1^).^40^ Olefinic (alkenes) angular deformation C=C (at 992 and 909 cm^–1^) with a low intensity was ascribed to the unsaturations in the alkyl side chains of cardol, methyl cardol, and cardanol, suggesting the lower content of these compounds, compared to that of anacardic acid;^30,34^ these facts were attributed to the low temperature (38 °C) during the nutshell drying process, and thus, the content of the formed cardanol was minimized. The bands at 900 to 625 cm^–1^ could be associated with the C–H out-of-plane bending vibration, due to the substitutions in the phenolic ring;^41,42^ the bands at 646, 771, and 820 cm^–1^ were ascribed to the 1, 3 meta-disubstituted ring while that at 820 cm^–1^ was ascribed to ortho-disubstituted ring.

### 3.2 ML Measurements

Table 1 lists the ML of the S135 steel samples as a function of the inhibitor (EES) concentration (*C*_i_), the degree of surface steel coverage (θ) by the EES, and its protective efficiency, based on the ML (% IE_ML_), measured after 24 h of steel immersion in the SME solution. The results revealed that the ML decreased ≈7 times with the increase of the EES concentration, and this fact was attributed to the increment in the EES surface coverage value (θ), associated with better adsorption of EES molecules on the steel surface, thus protecting it from the aggressive simulated marine environment (SME). The maximum % IE_ML_ was 86% at a 150 mg L^–1^ EES inhibitor concentration.

**Table 1:** ML of Carbon Steel S135, EES Surface Coverage (θ), and Inhibitor Efficiency, after 24 h of Immersion in the SME

*C*_i_ (mg L^–1^)	ML (mg)	θ	% IE_ML_
control	35.0		
25	10.04	0.71	71
50	8.00	0.77	77
100	6.02	0.83	83
150	5.01	0.86	86

### 3.3 Adsorption Isotherm

**Figure 3:**
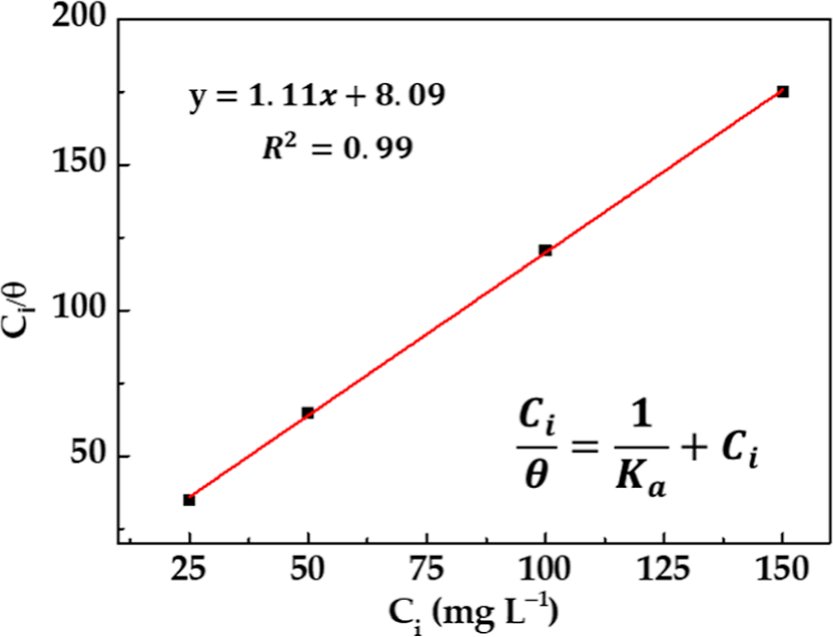
Langmuir adsorption isotherm of the EES inhibitor for the S135 carbon steel surface, exposed to the SME at 21 °C.

The nature of the physicochemical interaction inhibitor–metal surface could be described using the adsorption isotherm, plotting the θ data (Table 1) as a function of *C*_i_. In this study, the best fit (*R*^2^ = 0.998) was obtained for the Langmuir isotherm (Figure 3 and eq 4), suggesting that a monolayer of the EES inhibitor molecules was adsorbed.^43^
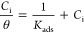
4

The steel surface was considered homogeneous, assuming it as such after the pretreatment of the steel surface (polishing and cleaning from impurities), allowing reasonable homogeneous distribution of the adsorption sites. The value of the Langmuir adsorption isotherm slope (Figure 3) was almost equal to the unity (=1.11), suggesting that there is an interaction (intermolecular repulsion or attraction) between the inhibitor molecules, causing a slight deviation of the slope from the unity.

The standard equilibrium constant of the adsorption and desorption process (*K*_ads_) was calculated from the Langmuir linear fit, and its value was used to determine the standard Gibbs free energy of adsorption (Δ*G*_ads_, eq 5)^44^
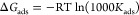
5where *R* is the universal gas constant (8.314 J mol^–1^ K^–1^), *T* is the temperature (295 K), and the constant 1000 g L^–1^ represents the concentration of the solvent molecules. The value of Δ*G*_ads_ = −28.82 kJ mol^–1^ is an indication of spontaneous physisorption (Δ*G*_ads_ > −20 kJ mol^–1^) and chemisorption (Δ*G*_ads_ < −40 kJ mol^–1^) processes of the EES inhibitor on the steel surface.^45^ Thus, the inhibitor mechanism may be attributed to electrostatic van der Waals interaction and electron transfer between the rich electron sites (benzene ring and carboxylic group) of the EES molecules and the steel. The adsorption of organic molecules of the *marañon* nutshell extract could occur because of their hydrophilic sites’ (substituted benzene ring, Figure 1a) interaction with the steel S135 surface, while the neutral charge of the hydrophobic sites (alkyl side chain) would act by repelling the aqueous SME aggressive solution away from the steel surface.^46^ It is reported that the unsheared pair electrons of the oxygen atoms of the carboxylic group –COOH (present in the anacardic acid, with a salicylic acid structure) and the π electrons of the aromatic rings are responsible for the adsorption of the *marañon* nutshell extract molecules. Additionally, the –OH in the benzene ring is considered as an electron donor group, which could increase the electron density of anacardic acid.^47^ It has also been suggested that anacardic acid may act as an antioxidant^48^ because of its high selectivity toward Fe^2+^ and Fe^3+^ ions, chelating them.^49^ Thus, if the Fe (carbon steel S135) surface tends to corrode, at the local anodic sites, the released Fe^2+^ and Fe^3+^ ions will be chelated by anacardic acid, and at these anodic sites, the formation of a new Fe-oxide corrosion layer will be suspended (diminishing of the corrosion rate).^50^ According to the quantum chemical calculation report,^51^ the structure of ortho-substitution in the benzene ring (as in the anacardic acid) is the reactive form arrangement with a low energy gap, presenting a higher value of overall softness (σ), which is an indicator of the ability to lose or receive electrons, depending on the cationic or anionic form of the molecule. On the other hand, the molecular electrostatic potential map of salicylic acid^51^ suggests that in such a structure, the interaction with the positive Fe^2+^ and Fe^3+^ ions would occur through the unsheared pair electrons of the oxygen. Thus, these facts will contribute to the successful corrosion inhibition effect.

### 3.4 Open-Circuit Potential Measurement

**Figure 4:**
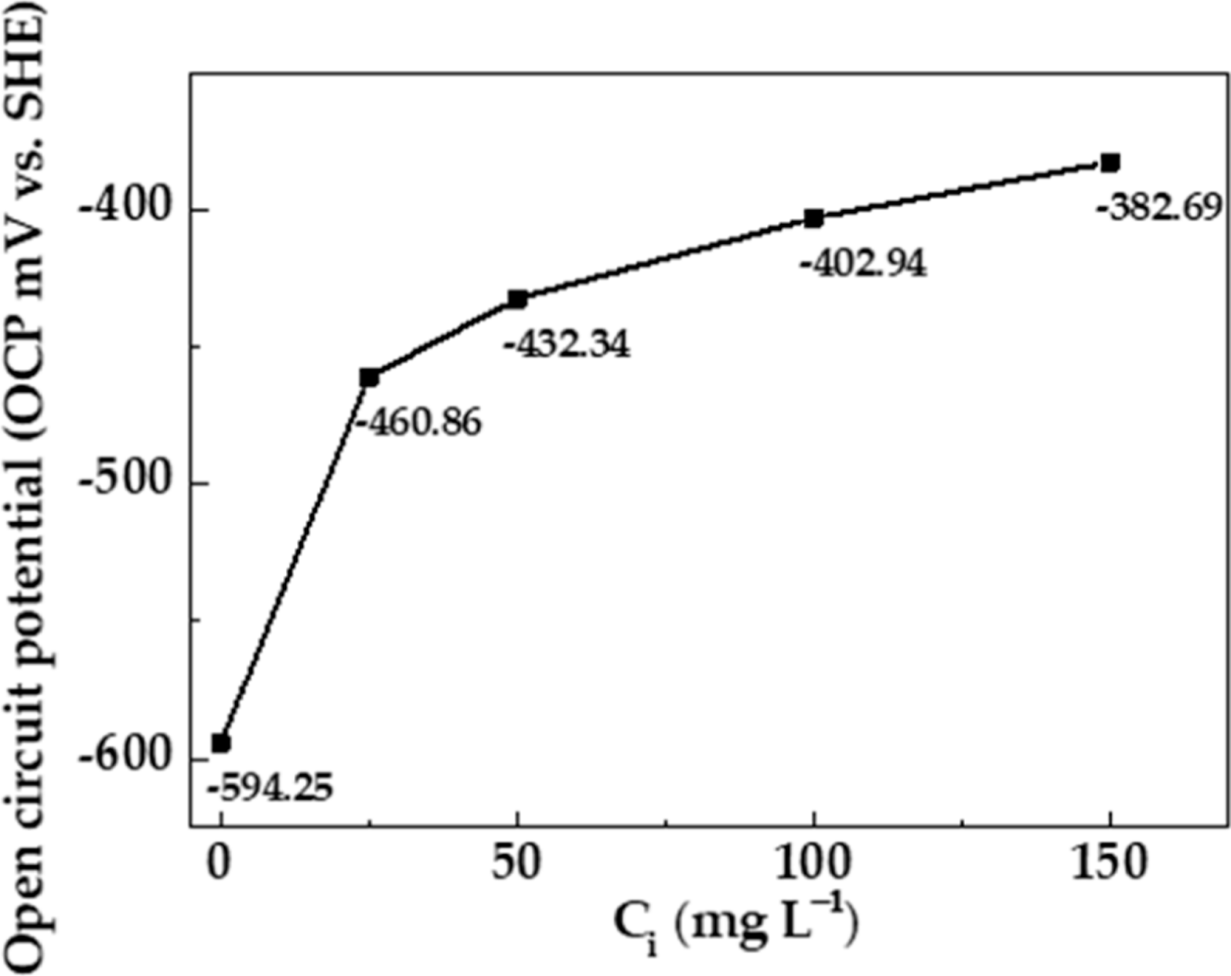
Change of the OCP of S135 steel, exposed to a simulated marine environment (SME), as a function of the EES inhibitor concentration.

The change in time of the free corrosion potential value of metal (at an open circuit, without an external polarization, OCP), during the metal exposure to aggressive environments, is a criterion that can indicate the variance in the metal corrosion resistance because of the formed layers on the metal surface and their composition and morphology. The OCP value is dependent on the metal activity (redox potential) and the surface homogeneity, as well as the aggressivity and electrolyte solution chemistry, with which the metal is in contact. Figure 4 presents the OCP values of the carbon steel S135, during its exposure to the SME, as a function of the *C*_i_ (inhibitor concentration). In the absence of an inhibitor, the OCP was ≈−594 mV, and then it shifted to less negative values, reaching −382 mV at *C*_i_ = 150 mg L^–1^. The last fact indicates that the EES may act as an inhibitor for the steel interface because of EES adsorption (Figure 3), attributed mostly to the anacardic acid ion-capturing chelating effect for the released Fe-ions (during the corrosion process). The change of the measured OCP values agrees with the tendency of steel ML decrease and EES surface coverage (θ) increase, based on *C*_i_ (Table 1).

### 3.5 Zeta Potential

The zeta potential value of the effective EES electric charge was ≈−5.15 mV, measured for 100 μL of EES added to 1 mL of SME (the model marine solution). Zeta potential is commonly used to quantify the net surface charge of nanoparticles dispersed in the bulk fluid, and in a NaCl solution (SME), they tend to agglomerate.^2,50^ The negative zeta potential was attributed to the molecules of the *marañon* nutshell extract, dissolved in the EES, since the nonionic surfactant “Tween 20”, as a part of the EES, did not acquire any charge in the SME solution. According to the literature, when the surfactant is above the critical micelle concentration (CMC ≈ 40–152 μM) in water solution, at room temperature,^51,53^ the self-assembly of micelles occurs and they are considered as effective carriers for hydrophobic and hydrophilic compounds (depending on the arrangement of the micelles).^52,54^ Due to the negative zeta potential of the *marañon* nutshell extract dissolved in the EES, it could attract the positively released Fe^2+^ and Fe^3+^ once the steel surface initiates to corrode and capture them in chelates, blocking the active steel anodic sites for new corrosive attacks.

### 3.6 Surface Characterization: Optical Microscopy and XPS Analysis

**Figure 5:**
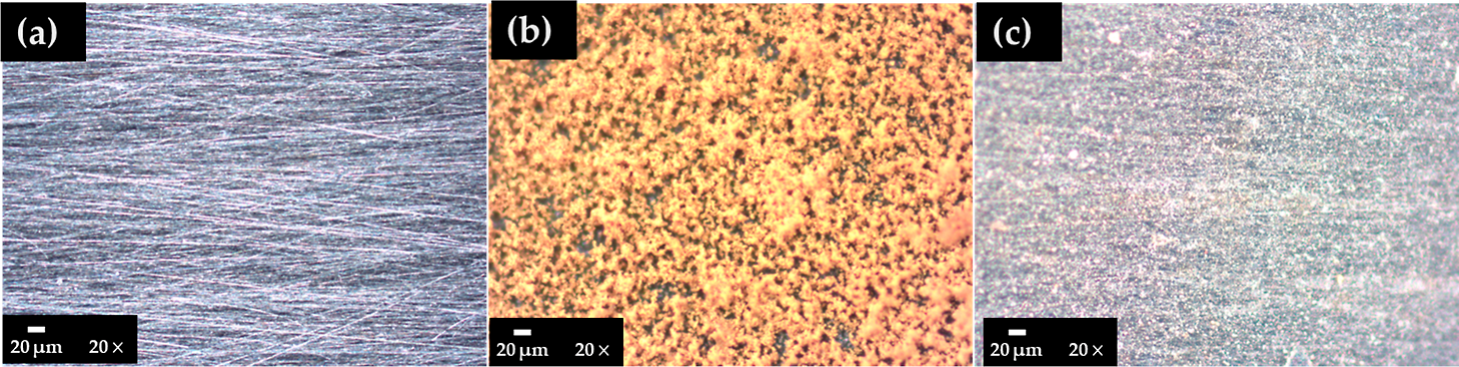
Optical microscopy images of the S135 steel surface (a) before and after 24 h of exposure to the SME: (b) in the absence of the EES inhibitor and (c) with 150 mg L^–1^ EES.

The optical images compare the steel surfaces before the immersion test in the SME (Figure 5a), after exposure for 24 h in the absence of the EES inhibitor (Figure 5b), and in the presence of 150 mg L^–1^ inhibitor (Figure 5c). The localized corrosion attacks are observed on the steel surface in the absence of the inhibitor (Figure 5b) compared to that in the presence of the EES inhibitor (Figure 5c).

**Figure 6:**
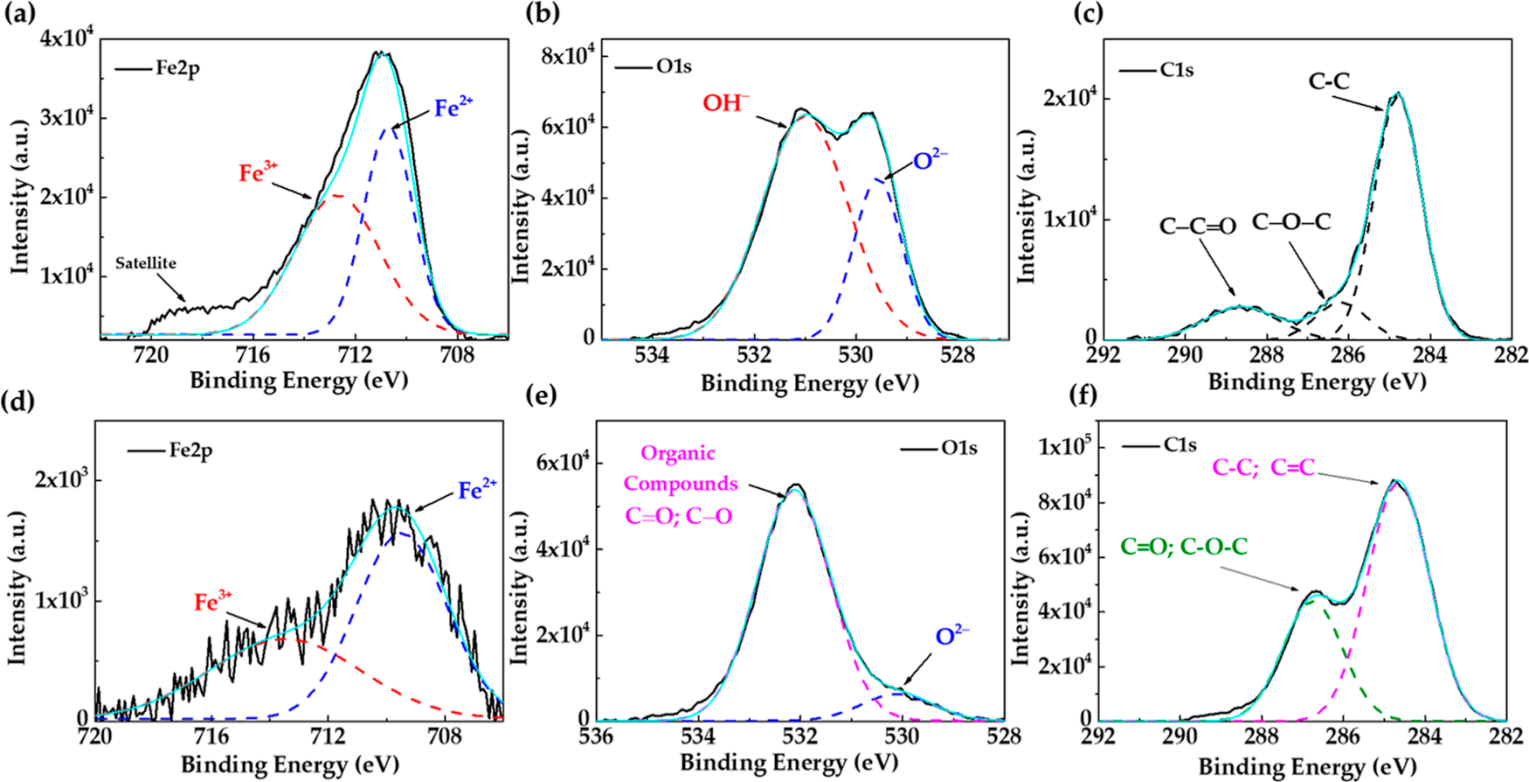
XPS spectrum of the peaks Fe 2p, O 1s, and C 1s presented in the S135 steel control sample (a–c); S135 at a 150 mg L^–1^ concentration of the EES inhibitor in the SME solution (d–f).

Figure 6 compares the XPS spectra of the S135 steel surface (control sample) after exposure to the SME in the absence of the EES inhibitor (Figure 6a) and with the addition of 150 mg L^–1^ EES to the solution SME.

In the absence of the inhibitor, the deconvolution of the Fe 2p (Figure 6a) of S135 surface shows the characteristics peaks of the Fe^2+^ (710.5 eV) and Fe^3+^ (712.5 eV) states, while for O 1s (Figure 6b), the presence of O^2–^ (529.6 eV) and OH^–^ (531.1 eV) is indicated: these peaks suggest that the Fe oxides/oxyhydroxides, as steel corrosion products, have been formed on the surface during its exposure to the aggressive marine environment; the optical microscopy image of that surface (Figure 5a) shows the localized corrosion attacks, influenced by the presence of Cl^–^ ions. According to the literature, the most common corrosion products consist of Fe-oxyhydroxides in their different crystalline structures, such as goethite (α-FeOOH) and lepidocrocite (γ-FeOOH) with an orange and brown color.^55^ In addition, the formation of Fe_3_O_4_ with mixed iron Fe^2+^ and Fe^3+^ states could form in marine environments.^56^ The C 1s of the control sample (Figure 6c) is associated with the presence of carbon contamination, such as CO_2_ from the air environment.

On the other hand, in the presence of the EES inhibitor in the SME, the XPS spectra of the S135 carbon steel present one order of decrease of the Fe 2p peak intensity (Figure 6d), attributed to Fe^2+^ (709.7 eV) and Fe^3+^ (712.8 eV); the peak associated with O^2–^ (530.0 eV, Figure 6e) diminished significantly in intensity; this reduction was attributed to the chelating effect of the Fe^2+^ and Fe^2+^ by anacardic acid, thus leading to the reduction of Fe corrosion product formation. The peak at 532 eV was ascribed to the hydroxyl (–OH) groups of the alkylphenols organic compounds of the *marañon* nutshell extract in EES.^57,58^ In the presence of the inhibitor, the intensity of the peak of C 1s (Figure 6f) changed, which suggested the presence of an organic adsorbed layer: the peak at 284.8 eV was attributed to C–C and C=C bonds of the benzene rings^59^ while that at 286.5 eV was ascribed to C=O (in −COOH) and C–O–C.^60^ The XPS spectrum (Figure 6) collaborates with the FTIR analysis of the *marañon* nutshell extract (Figure 2).

### 3.7 Nyquist and Bode Electrochemical Impedance Diagrams (EIS)

**Figure 7:**
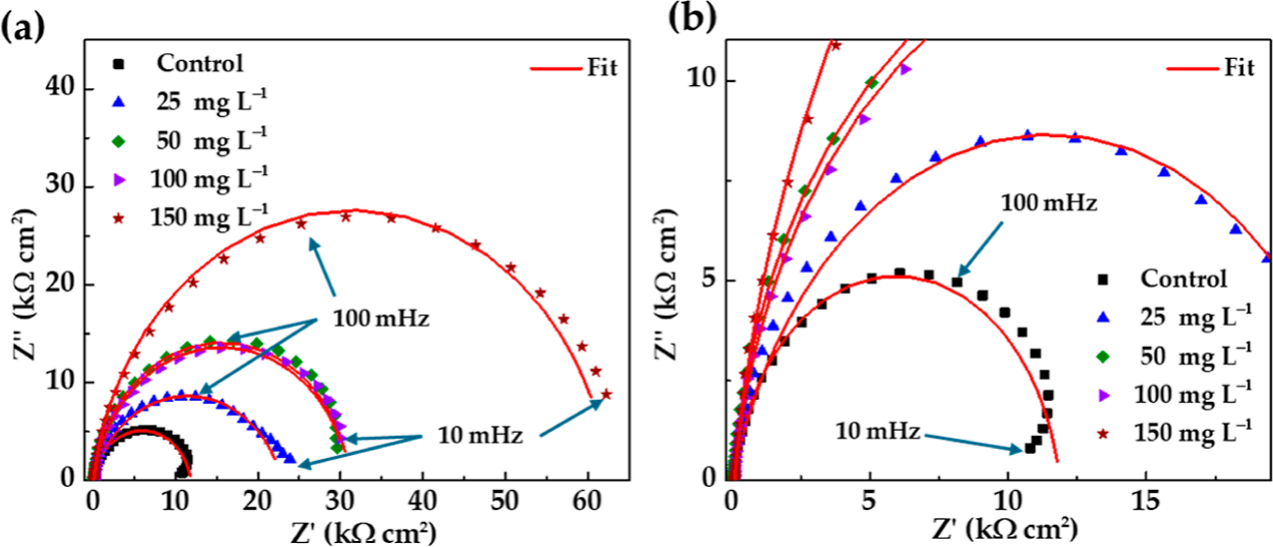
Nyquist impedance diagram of the carbon steel S135 after 24 h of exposure to the simulated marine environment (SME) in the presence of different concentrations of the EES inhibitor (a) and zoom image (b).

Figure 7 presents the Nyquist diagrams of EIS for carbon steel S135 after 24 h of exposure to the simulated marine environment (SME), with the addition of different concentrations of the EES inhibitor extract. The diagram of the control sample (in the absence of an inhibitor) showed a capacitive loop at the high-frequency domain (100 kHz to 100 mHz), followed by an inductive arc at the low-frequency domain (10–100 mHz). The inductive arc could be associated with the formation of unstable Fe corrosion products^61^ on the steel surface (Figure 5). In the presence of the EES inhibitor, the inductive arc disappears, and single depressed incomplete semicircles are observed, corresponding to the charge-transfer control of the corrosion process.^62^ The diameters of the incomplete semicircles increase with the increment of the EES concentration, which is an indication of the hindering of the corrosion process.^63,64^

**Figure 8:**
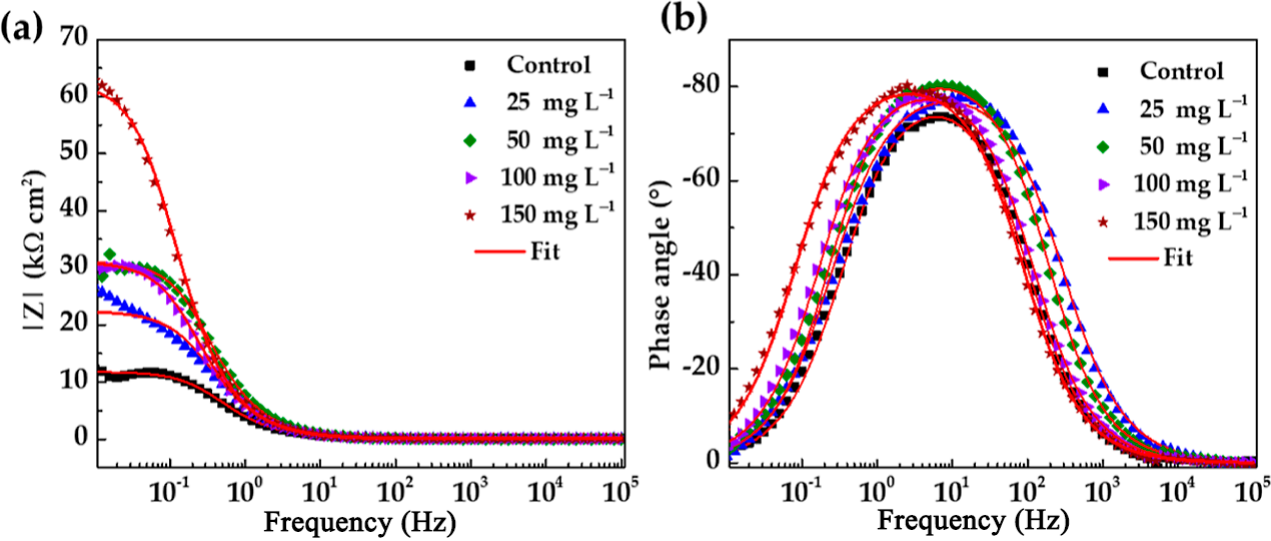
(a) Module of impedance |*Z*| and (b) phase angle of the Bode impedance diagrams of the carbon steel S135, after 24 h of exposure to the simulated marine environment (SME), in the presence of different EES inhibitor concentrations.

The module of impedance |*Z*| and phase-angle Bode diagrams of EIS (Figure 8) showed that the |*Z*| tended to increase and reached kΩ cm^2^ at a 150 mg L^–1^ EES concentration (Figure 8a), as an indication of higher inhibitor efficiency. Meanwhile, the phase angle (Figure 8b) revealed values of ≈−10° at low frequencies (for 150 mg L^–1^ EES), while at the medium frequencies, the phase angle was ≈−80°, when the adsorption process softened on the steel surface.^65^

**Figure 9:**
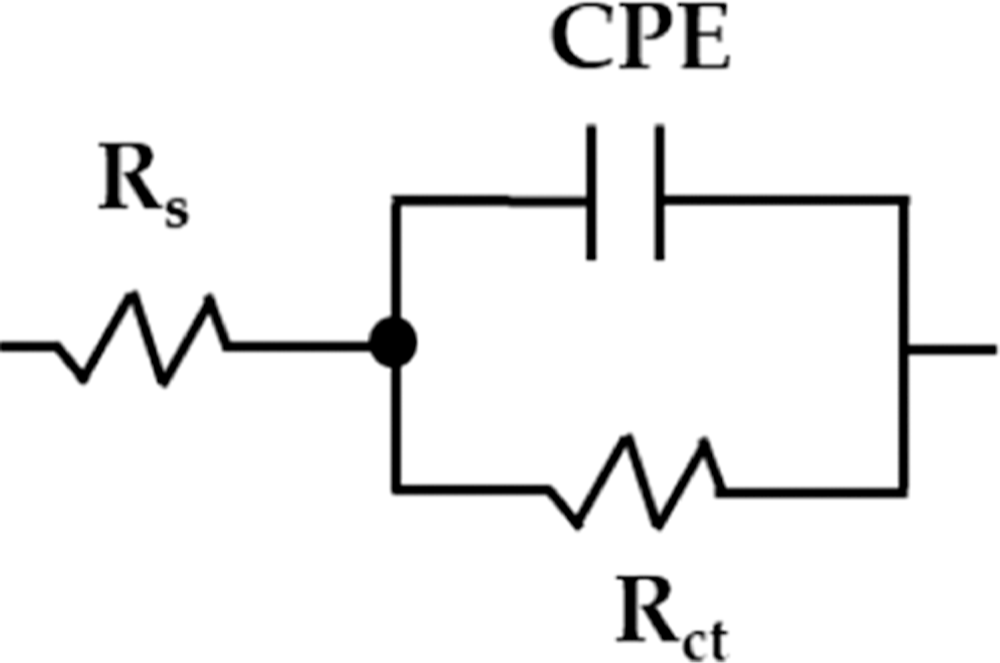
Simplified Randles equivalent circuit.

The phase-angle Bode diagrams (Figure 8b) showed only one phase maximum, revealing that the corrosion process occurred through one step, corresponding to one time constant. To quantify the EIS data, an equivalent electrical circuit is used, known as the simplified Randles circuit (Figure 9),^64,65^ which consists of the *R*_s_ (solution resistance), and a CPE constant phase element, which is used instead of pure capacitance of the electrical double layer at the metal–electrolyte interface, to consider the roughness and heterogeneity of the carbon steel substrate,^64,66^ connected in parallel with the *R*_ct_ (charge transfer resistance).^65^

Table 2 lists the fitted parameters obtained from the EIS measurements, for which the goodness of fit (χ^2^) was good in most cases (10^–4^). The values of the *n* parameter were relatively constant ≈0.90, as a function of the EES inhibitor concentration, ascribed to the capacitive nature of the passive film formed on the steel surface, as well as to the reduction of the surface inhomogeneity due to the adsorption of inhibitor molecules.^68^ The polarization resistance (*R*_p_), as an almost equivalent of the *R*_ct_ values minus the *R*_s_,^61^ was used as an indicator of the hindrance of the corrosion process of the carbon steel exposed to the model marine solution. When the EES inhibitor molecules are adsorbed on the steel surface, they may act as a physical barrier due to the hydrophobic properties of the alkyl side chains of the *marañon* nutshell extract molecules, displacing the water molecules and reducing the electrical conductivity of the outer layer formed on the carbon steel surface. These effects lead to an increase in the *R*_p_ value of ≈5.30 times (at 150 mg L^–1^ of EES), compared to that of the control steel sample in the absence of the EES inhibitor. Meanwhile, the % IE_EIS_ reached 81.15% (Table 2). EIS fitted data of the S135 carbon steel, after 24 h of exposure to a SME at different concentrations of the inhibitor, are presented in Table 2.^67^

**Table 2:** EIS Fitted Data of S135 Carbon Steel after 24 h of Exposure to a Simulated Marine Environment (SME), as a Function of the EES Inhibitor Concentration

steel	*C*_*i*_ (mg L^–1^)	*R*_s_ (kΩ cm^2^)	*R*_p_ (kΩ cm^2^)	CPE (μSs^*n*^ cm^–2^)	*n*	χ^2^ (10^–4^)	% IE_EIS_	θ
S135	0	0.065	11.79	42.62	0.89	7.78		
	25	0.037	22.45	29.68	0.90	5.25	47.48	0.47
	50	0.057	31.02	22.29	0.93	6.32	61.99	0.61
	100	0.073	31.08	30.76	0.93	4.21	62.06	0.62
	150	0.090	62.57	29.62	0.93	4.83	81.15	0.81

The effective capacitance values (*C*) were calculated from the CPE values, according to the Brug formula (eq 6),^69^ and the values were related to the Fe-oxide layer thickness (*d*) formed on the S135 steel surface (eq 7),^70,71^ where ε_0_ is the vacuum permittivity (8.85 × 10^–14^ F cm^–1^), A is the working area (cm^2^), and ε is the dielectric constant of the Fe-oxide layer ≈12 for carbon steel.^72^
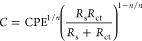
6
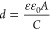
7

**Figure 10:**
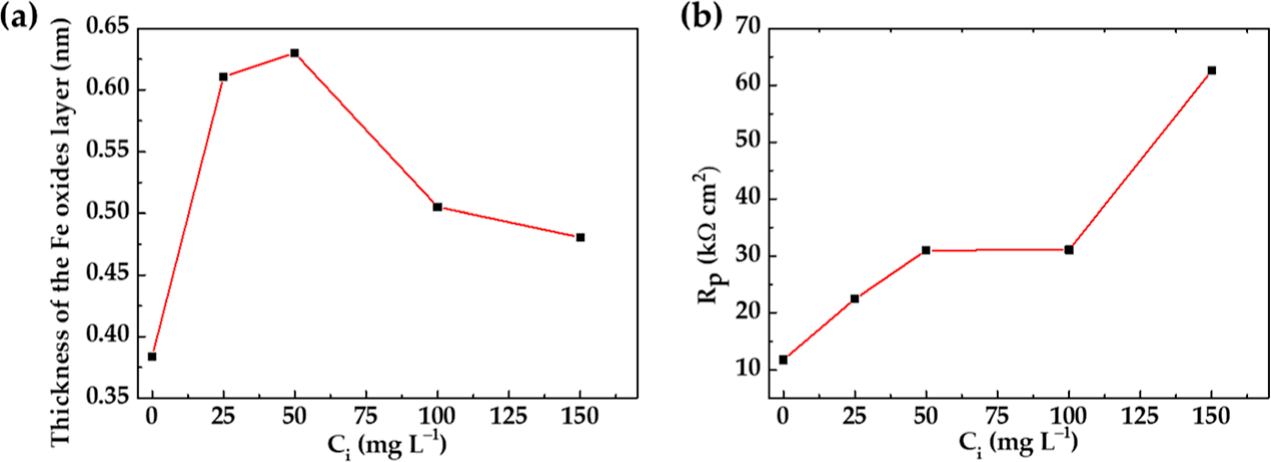
Thickness of the Fe-oxide layer (a) formed on the S135 steel surface and its *R*_p_ values (b) as a function of *C*_i_, corresponding to 24 h of exposure to the SME.

Figure 10 compares the change of the thickness of the Fe oxide layer *d* formed on the steel surface (Figure 10a) and its *R*_p_ values (Figure 10b), influenced by the EES inhibitor concentration. *d* of the steel substrate (control sample) had the lowest value, attributed to the formation of an unstable Fe-corrosion process, during the oxide and/or hydroxide layer formation, accompanied by the Fe-ion release at the active anodic sites of the iron matrix (carbon steel surface) immersed in the aggressive environment SME, indicated by the inductive arc in the Nyquist diagram (Figure 7). At the concentrations of 25 and 50 mg L^–1^, the *d* increases, while the % IE_EIS_ ≈ 61% (Table 2), attributed to the gradual capture of the released Fe^2+^ and Fe^3+^ ions and chelating them by anacardic acid, thus blocking the active steel anodic sites for new corrosive attacks. Once the concentration of the EES inhibitor reached 100 mg L^–1^, the thickness of the corrosion layer decreased, as well as the metal ML tended to have a lower value (Table 1), as an indication of a retarded corrosion process. The observed changes in the thickness of the layer formed on the steel as a function of the EES inhibitor concentration are also reflected in the increasing values of the polarization resistance (Figure 10b). The highest *R*_p_ value corresponds to the highest concentration of EES (150 mg L^–1^) while that of the control sample is ≈6 times lower.

## 4 Conclusions

The natural *marañon* nutshell extract (EES) was studied as an inhibitor for S135 carbon steel, exposed to a simulated marine environment (SME), when an aqueous layer at 100% air humidity was formed on the metal surface. The inhibitor effect was deduced by a variety of different methods (ML, adsorption isotherm, zeta potential, corrosion open circuit potential, and EIS Nyquist and Bode diagrams) and steel surface analysis (XPS and FTIR spectroscopy and optical microscopy images).

The FTIR analysis suggested that anacardic acid is the main component of the *marañon* nutshell extract (due to the low temperature used in the dry process), while the functional groups of alkyl phenols (cardol, cardanol, and methyl cardol) were detected at a lower intensity than those of anacardic acid.

The ML of the steel decreases when an EES inhibitor is added to the SME, reaching an inhibitor efficiency of ≈86% at a 150 mg L^–1^ concentration, accompanied by an increase in the EES surface coverage (θ). The adsorption of the inhibitor followed the Langmuir isotherm. According to Δ*G*_ads_ = −28.82 kJ mol^–1^, the EES inhibitor may protect the steel surface due to its van der Waals physical–chemical adsorption, attributed to the molecule electrostatic interaction and electron transfer with the carbon steel surface.

It was considered an antioxidant activity of the EES inhibitor, which was mostly attributed to anacardic acid, capturing the Fe^2+^ and Fe^3+^ ions and chelating them, thus blocking sites for gradual corrosion progress. This consideration collaborated with the shift of the OCP-free corrosion potential in ≈200 mV to less negative values.

The *marañon* nutshell extract (dissolved in EES) acquired a negative zeta potential (−5.15 mV) when it was added to the SME, which suggested its affinity toward the Fe^2+^ and Fe^3+^ and their capture, once the steel surface initiated to corrode, thus blocking the active anodic sites and diminishing the steel corrosion rate. In the absence of an inhibitor, XPS analysis suggested that the corrosion layer was composed of Fe-oxides/oxyhydroxides. In the presence of the inhibitor, the optical microscopy images did not indicate the occurrence of corrosion attacks.

The EIS diagrams analysis revealed that the *R*_p_ polarization resistance of the steel surface increased ≈6 times in the presence of an EES inhibitor, compared to that of the steel control sample. The inhibitor efficiency based on the EIS analysis was ≈81% at a 150 mg L^–1^ EES concentration. The calculated thickness *d* of the Fe-oxide layer showed a reduction effect, which was attributed to retardation of the corrosion process in the presence of the EES inhibitor.
